# Grey and Black Anti-Hail Nets Ameliorated Apple (*Malus* × *domestica* Borkh. cv. Golden Delicious) Physiology under Mediterranean Climate

**DOI:** 10.3390/plants10122578

**Published:** 2021-11-25

**Authors:** Cátia Brito, Manuel Ângelo Rodrigues, Luís Pinto, Alexandre Gonçalves, Ermelinda Silva, Sandra Martins, Luis Rocha, Ivo Pavia, Margarida Arrobas, António Castro Ribeiro, José Moutinho-Pereira, Carlos M. Correia

**Affiliations:** 1 CITAB—Centre for the Research and Technology of Agro-Environmental and Biological Sciences, University of Trás-os-Montes and Alto Douro, Quinta dos Prados, 5000-801 Vila Real, Portugal; cvqbrito@utad.pt (C.B.); agoncalves@morecolab.pt (A.G.); ermelinda.silva@blc3.pt (E.S.); scpmartins@utad.pt (S.M.); luis.rocha@blc3.pt (L.R.); ivo.mmp@gmail.com (I.P.); moutinho@utad.pt (J.M.-P.); 2 CIMO—Centro de Investigação de Montanha, Instituto Politécnico de Bragança, 5300-252 Bragança, Portugal; angelor@ipb.pt (M.Â.R.); lpinto@morecolab.pt (L.P.); marrobas@ipb.pt (M.A.); antrib@ipb.pt (A.C.R.); 3 MORE—Collaborative Laboratory Mountains of Research, Brigantia Ecopark, 5300-358 Bragança, Portugal; 4 Association BLC3—Technology and Innovation Campus, Centre Bio R&D Unit, Rua Comendador Emílio Augusto Pires, 14, Edifício SIDE UP, 5340-257 Macedo de Cavaleiros, Portugal

**Keywords:** glomalin-related soil proteins, photosynthesis, water relations, foliar metabolites, minerals, yield

## Abstract

The use of anti-hail nets on orchards changes the microclimate underneath the net. This might be of great importance in apple growing regions characterized by high radiation levels and hot and dry climates during the summer season. But, depending on the net colour and on the local climatic conditions, the shade promoted triggers different responses by the trees. Grey and black anti-hail nets were applied in an apple orchard (cv. ‘Golden Delicious’) located in Northeast Portugal. Under the nets a lower concentration of glomalin related-soil proteins was observed, along with an improvement on trees water status, stomatal conductance, net photosynthetic rate, total chlorophylls, N, Mg, Fe and Cu concentrations, as well as an increase in mean fruit weight. The major difference between nets was on the photosynthetic efficiency, being higher on black net in sunny days, while grey net performed better under cloudy conditions. The use of netting systems proved to be effective in improving “Golden Delicious” apple trees performance under a Mediterranean climate, mainly when the radiation reaching the plants surpass the tree saturation point for photosynthesis. Therefore, these findings anticipate solutions for current and forecasted negative effects of climate change.

## 1. Introduction

The domestic apple tree (*Malus* × *domestica* Borkh.) is one of the most cultivated fruit crops worldwide, after *Citrus* and grapevine [1]. Usually, it is cultivated in temperate regions or at high altitudes but, although the climatic conditions of such regions may result in higher fruit production, these areas are frequently affected by hailstorms [2]. Moreover, some commercial apple growing regions are also characterized by high light intensity, low rainfall and high temperatures [3]. Excessive solar radiation, heat and/or drought stresses can be detrimental to the trees due to higher oxidative damage, photorespiration and photoinhibition [4,5], which ultimately might also reduce productivity. Furthermore, damages to the fruit caused by hailstorms [6], the development of sunburn in fruit due to excessive solar radiation [7] and high sensitiveness of fruit growth to drought [8,9] may cause huge economic losses for growers. Farmers and researchers searched for practical solutions to protect orchards, the netting systems being one of the most promising. Netting is the most effective method to protect from hailstorms and to reduce the intensity of the transmitted light and wind speed [2,3,6,10,11,12], which may cause changes on orchard’s microclimate, including the reduction in air, leaf, fruit and soil temperature. In addition, nets reduce evapotranspiration and increase soil moisture, which, in turn, increases soil water availability and reduces irrigation costs [3,9,13,14]. Therefore, nets might be of great importance in the context of climate change, particularly in conditions of extreme events, as the case of hailstorms and heat waves [15]. 

Changes induced by nets in orchard microclimate might also affect soil microbial communities, as arbuscular mycorrhizal fungi (AMF), which produce fungal proteins quantified from soil as glomalin-related soil proteins (GRSP) [16,17]. GRSP have received increasing attention, for their role in soil aggregate stability, protection from soil erosion, carbon storage, soil fertility improvement, reduction of greenhouse gases emissions and stress tolerance enhamcement [16], still the effects caused by the use of nets on orchard ecosystem need to be further understood. The induced changes in orchard’s microclimate can also affect tree physiology, ionome, growth, yield and fruit quality, in a level depending on the local climatic conditions, the degree of shading, the colour and aperture size of the net, the cultivar/rootstock, among others [6,10,11,12,18,19,20,21,22]. Unfortunately, data currently available on netting influence on trees physiological responses are usually contradictory. Anti-hail nets of different colours (black, white, green, and red) change light quality and the levels of photosynthetic active radiation (PAR), reducing the intensity [6,11,12,18,23], which in turns might influence trees physiological processes in a different extent. Previous studies reported opposite responses in terms of photosynthetic activity [10,19,24], as the influence on photosynthesis depends on the relation between the incident radiation and the light saturation point of plant genotypes. Under saturating light conditions, either by the excess of light and/or by other environmental stresses, net shading could benefit plants, as it reduces light-induced damages and, thus, photoinhibition [20]. In addition, the improvement of plant water status was also described [20,25] and, as a typical shade response, leaf chlorophyll concentration was higher, while leaf thickness was lower under the nets [6]. Neverthless, Bosco et al. [10] found no influence of netting on leaf chlorophyll concentration of both ‘Royal Gala’ and ‘Fuji Suprema’ cultivars, while in “Royal Gala” a reduction in leaf thickness was recorded. Solomakhin and Blanke [19] reported the occurrence of shade avoidance responses in apple trees under hailnets, namely smaller trunk diameter but larger number and longer bourse shoots. Regarding the effect of nets on the concentration of macro and micronutrients in the leaves and its seasonal variation, the available data is currently little [21]. As nutrient uptake and the subsequent distribution within the plant, change according to plant needs and in response to environmental conditions [26], the influence of nets on nutrient dynamics deserves further attention. The shading influence on yield and/or fruit size also varies largely in response to a range of factors, being the most determinants the net colour and environmental conditions [2,6,9,18,20,21,22,27].

To properly deal with environmental constraints and to develop new irrigation strategies in the netting systems, more research is needed to provide reliable information for different species and/or cultivars and geographical/climatic locations [11]. ‘Golden Delicious’ is one of the main apple cultivars worldwide and, as far as we know, no studies addressed the anti-hail net influence on soil microbiological communities and few accessed trees’ physiology. Therefore, we hypothesized that, by changes induced in orchards microclimate, the nets shading might affect soil microbiology and ‘Golden Delicious’ apple trees physiological responses, that in turns will help to explain potential differences in yield. Based on these hypotheses, the objective of this study was to characterize the influence of a grey and a black anti-hail net in glomalin related soil proteins, leaf water relations, photosynthetic responses, leaf metabolomics, ionome and crop yield.

## 2. Results

### 2.1. Glomalin-Related Soil Proteins

The values of total and easily extractable glomalin-related soil proteins (GRSP) were significantly higher in the plot kept uncovered in comparison with those that received the anti-hail nets, whereas no significant differences were found in GRSP between the plots covered with the grey net and black net (Table 1).

### 2.2. Leaf Gas Exchange and Chlorophyll a Fluorescence

**Figure 1:**
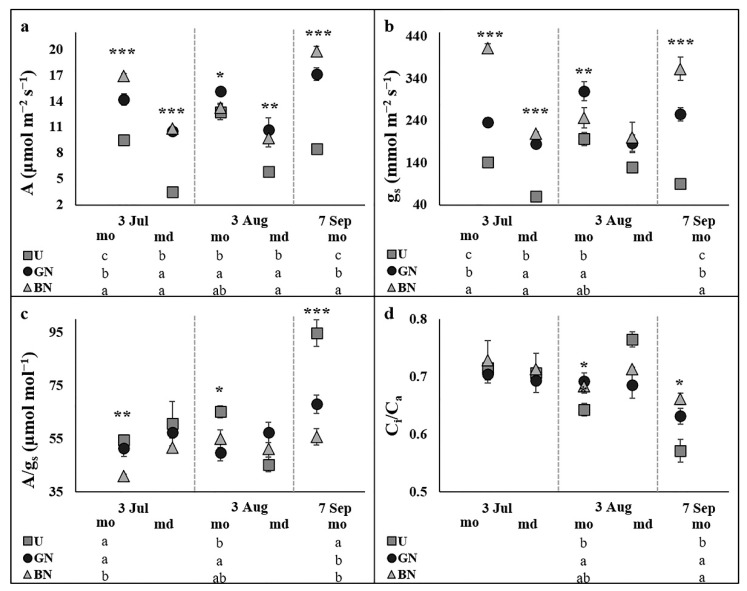
Evolution of leaf gas exchange parameters in plants uncovered (U), under grey net (GN) and black net (BN) throughout the experiment during the morning (mo) and midday (md) periods. Photosynthetic photon flux density values in the 5 sampling periods (from left to right) were 1418 ± 80, 1853 ± 55, 948 ± 41, 1917 ± 32 and 1390 ± 69 μmol photons m^−2^ s^−1^, respectively. Net photosynthetic rate (A, (**a**)), stomatal conductance (g_s_, (**b**)), intrinsic water use efficiency (A/g_s_, (**c**)) and ratio of intercellular to atmospheric CO_2_ concentration (C_i_/C_a_, (**d**)). Values are means ± SE. Different letters demonstrate significant differences between treatments in each analyzed date (* *p* < 0.05, ** *p* < 0.01, *** *p* < 0.001).

Figure 1 shows the leaf gas exchange response, where is evident the typical midday depression of A, reaching 63%, 26% and 36% on 3 July and 54%, 29% and 26% on 3 August for uncovered, grey and black nets treatments, respectively (Figure 1a). In general, the tendency of A and g_s_ were very similar, and consistently higher under the anti-hail nets than in the uncovered plants (Figure 1a,b). During the morning period of 3 July and 7 September the black net increased these variables in relation to the grey net, whereas during to the morning period of 3 August only the trees under the grey net stand out (Figure 1a,b). During the midday periods, both nets contributed to increase A (Figure 1a), while a significant and positive influence on g_s_ was only observed on 3 July (Figure 1b). The A/g_s_ and C_i_/C_a_ were only affected by the anti-hail nets during the morning periods (Figure 1c,d). The A/g_s_ was reduced by the black net on 3 July and 7 September, and by the grey net on 3 August and 7 September (Figure 1c). The C_i_/C_a_ was increased by the grey net on 3 August and both black net and grey net increased it on 7 September (Figure 1d).

**Figure 2:**
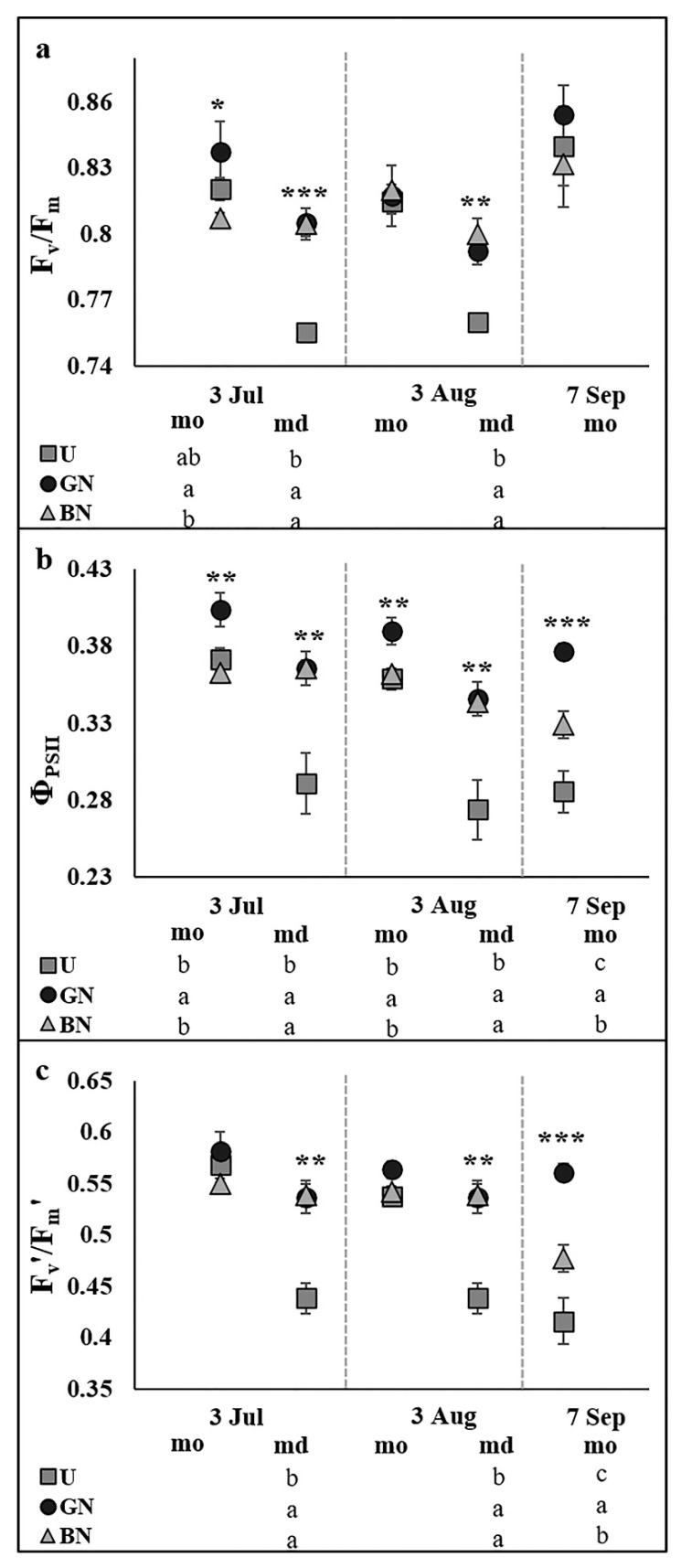
Evolution of leaf chlorophyll *a* fluorescence variables in plants uncovered (U), under grey net (GN) and black net (BN) throughout the experiment during the morning (mo) and midday (md) periods. Photosynthetic photon flux density values in the 5 sampling periods (from left to right) were 1418 ± 80, 1853 ± 55, 948 ± 41, 1917 ± 32 and 1390 ± 69 μmol photons m^−2^ s^−1^, respectively. Maximum quantum efficiency of PSII (F_v_/F_m_, (**a**)), effective quantum efficiency of PSII (Φ_PSII_, (**b**)) and capture efficiency of excitation energy by open PSII reaction centers (F_v_’/F_m_’, (**c**)). Values are means ± SE. Different letters demonstrate significant differences between treatments in each analyzed date (* *p* < 0.05, ** *p* < 0.01, *** *p* < 0.001).

Chlorophyll *a* fluorescence analysis revealed that, in general, the anti-hail nets positively influenced the photochemical reactions of photosynthesis, especially the grey net (Figure 2). Although grey net also contributed to increase the F_v_/F_m_ in the morning period of 3 July, this variable was mainly affected during the midday periods, where both grey net and black net contributed to increase it (Figure 2a). The Φ_PSII_ was increased by the black net during the midday periods monitored and the morning period of 7 September, while the grey net consistently increased this parameter in all the monitored periods, still standing out from the black net during the morning period of 7 September (Figure 2b). The F_v_’/F_m_’ was increased by both nets in the midday periods monitored and in the morning period of 7 September, where the grey net still stands out from the black net (Figure 2c).

### 2.3. Leaf Water Status and Sclerophylly Indexes

**Figure 3:**
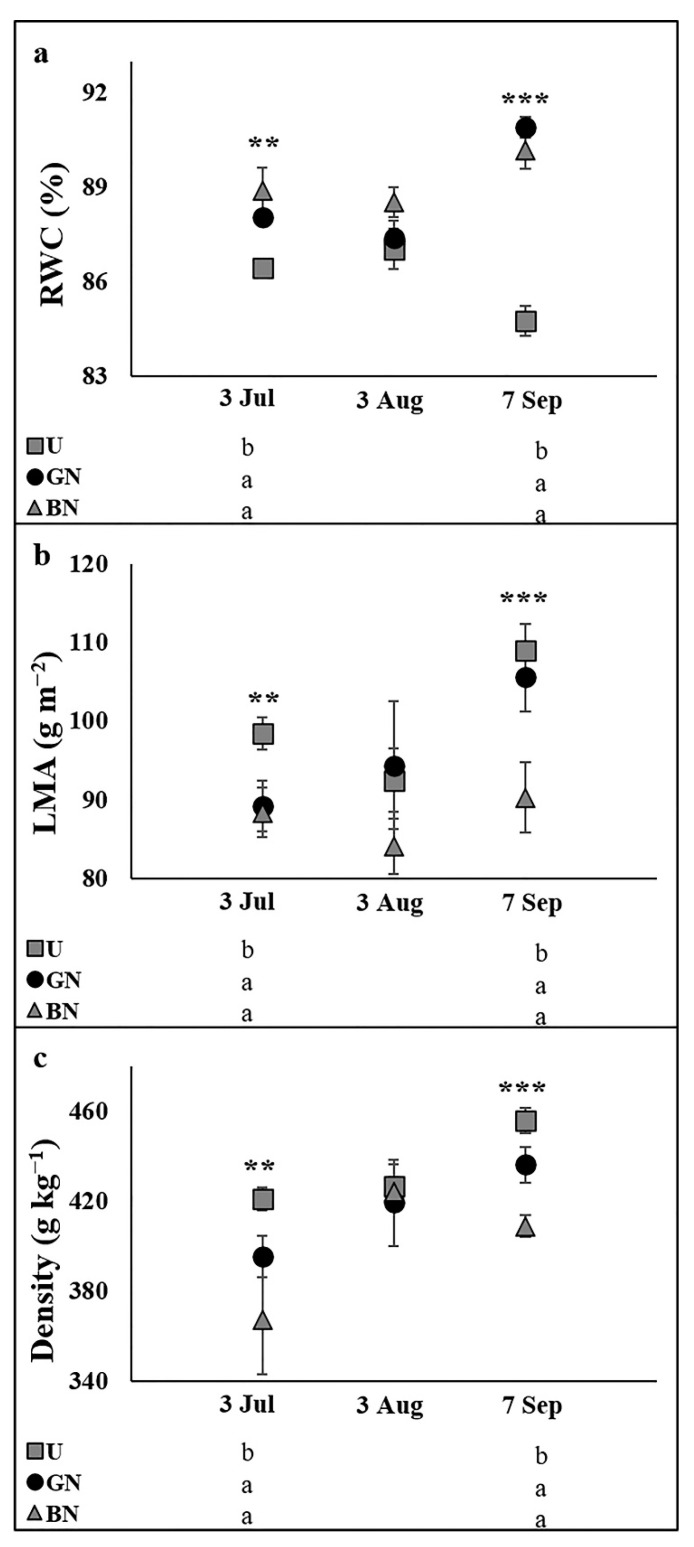
Evolution of leaf relative water content (RWC, (**a**)) and sclerophylly parameters (leaf mass area, LMA, (**b**), and density, (**c**) in plants uncovered (U), under grey net (GN) and black net (BN) throughout the experiment. Values are means ± SE. Different letters demonstrate significant differences between treatments in each analyzed date (** *p* < 0.01, *** *p* < 0.001).

Relative water content (RWC) was increased by the anti-hail nets on 3 July and 7 September, while on 3 August no significant influence was recorded (Figure 3a). LMA and leaf density was only affected on 7 September, where the black net contributed to reduce these variables (Figure 3b,c).

### 2.4. Leaf Biochemical Analysis

The influence of anti-hail nets in photosynthetic pigments and leaf metabolites concentration is presented in Table 2. Total soluble sugars (TSS), chlorophyll a/chlorophyll b ratio, total carotenoids (Car), total soluble proteins (TSP) and total thiols (-SH) concentrations were not significantly affected by the nets, while the concentration of total chlorophylls (Chl_(a+b)_) and the Chl_(a+b)_/Car ratio increased under the nets.

### 2.5. Leaf Ionome

**Figure 4:**
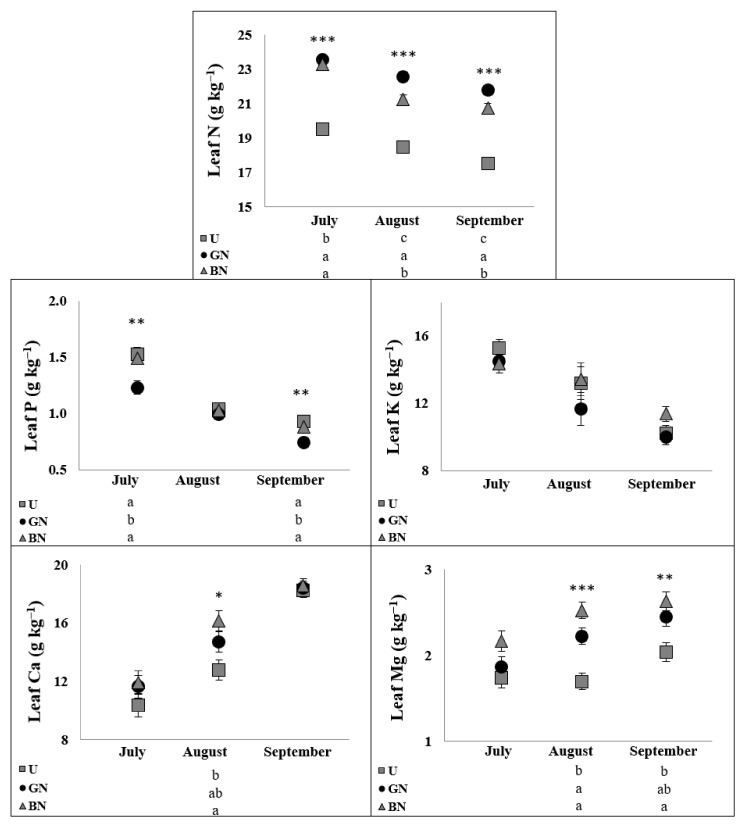
Evolution of macronutrients concentrations in apple leaves. Values are means ± SE. Different letters demonstrate significant differences between treatments in each analyzed date. (* *p* < 0.05, ** *p* < 0.01, *** *p* < 0.001).

**Figure 5:**
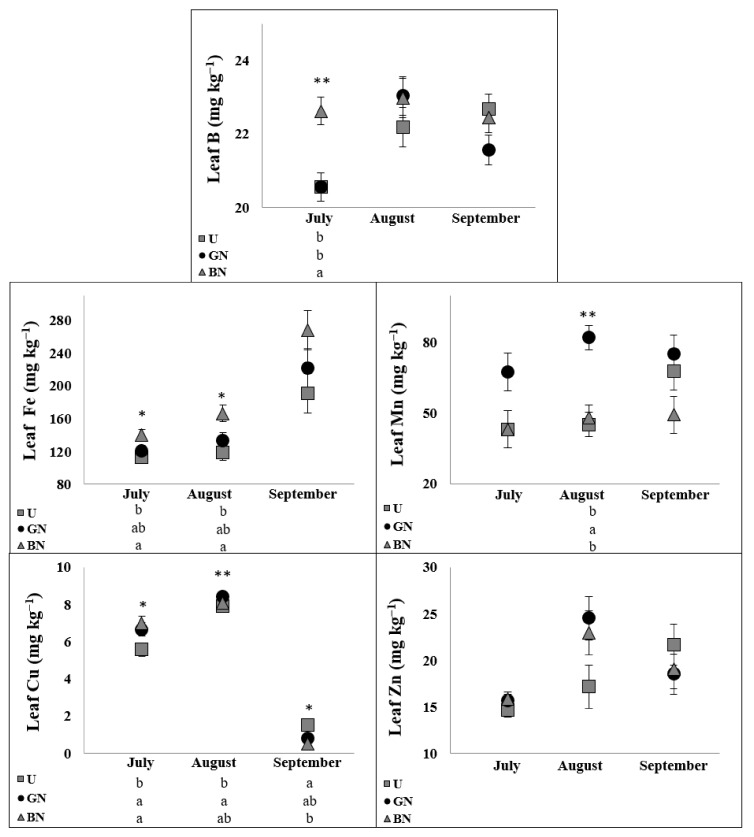
Evolution of micronutrients concentrations in apple leaves. Values are means ± SE. Different letters demonstrate significant differences between treatments in each analyzed date. (* *p* < 0.05, ** *p* < 0.01).

The indicators of tree mineral status during the summer (July, August and September) are presented in Figure 4 and Figure 5. The seasonal changes of leaf macronutrients concentrations revealed the decrease of N, K and P and the increase of Ca and Mg over the vegetative period (Figure 4). At the same time, both grey net and black net contributed to higher concentrations of N in all analysed dates and of Mg in the last sampling date. Meanwhile, P contents were improved by the grey net in June and September, while Ca was improved by the black net in August. Furthermore, while Fe and Mn increased over the vegetative period, B and Zn remained relatively stable (Figure 5). On the other hand, Cu showed an increase from July to August and then a decrease to September. Still, B contents were increased by the black net in July, Fe by the black net in July and August, Mn by the grey net in August and Cu by both nets in July, by the grey net in August and by the black net in September.

### 2.6. Trees Yield and Fruit Weight

**Figure 6:**
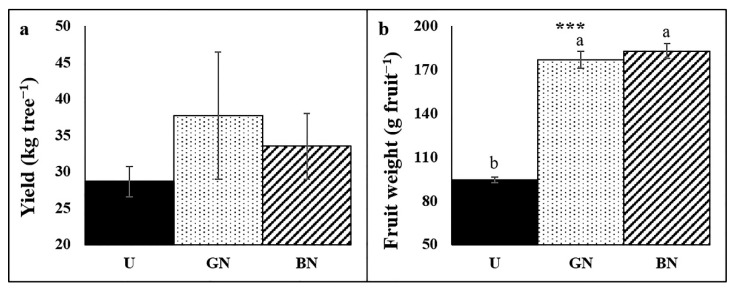
Yield (**a**) and mean of fruits weight (**b**) of plants uncovered (U), under grey net (GN) and black net (BN). Values are means ± SE. Different letters demonstrate significant differences between treatments (*** *p* < 0.001).

Although not statistically significant, an increase in yield in trees under the nets was recorded (Figure 6a), a tendency associated with the higher average fruit weight (Figure 6b).

## 3. Discussion

One of the primary benefits of protective netting is the reduction of solar radiation reaching the orchard environment underneath, modifying soil and atmospheric conditions [11]. The likely alleviation of stressful conditions, including low incident radiation, air and soil temperature and higher soil moisture [3,13,14], is somehow reflected in the lower values of total and easily extractable glomalin-related soil protein (GRSP) (Table 1). Some earlier studies reported that more unfavorable growing conditions induce the glomalin production by the arbuscular mycorrhizal fungi (AMF) as a stress-induced protein, being the functional roles of glomalin in soils a secondary consequence [28,29]. In fact, glomalin has been linked with heat shock proteins, a group of small proteins produced under environmental-related stress conditions [29,30]. The accumulation of GRSP in response to high temperatures was also previously reported [17,31].

One of the primary impacts of environmental stresses, whether drought, heat or high light, is the impairment of photosynthetic capacity, primarily by the reduction of g_s_ and, in more stressful conditions, also by damages caused in the photosynthetic apparatus [32]. Thus, the best photosynthetic performance by trees under netting (Figure 1) must be due to changes induced in the microclimate. The likely reduction in air temperature, evapotranspiration, vapor pressure deficit (VPD) and wind intensity [3,9,14,33] might contribute to higher soil water availability and decrease the driving force for transpiration, promoting an increase in g_s_ [34] and the CO_2_ input (Figure 1). Although under the nets the promotion of g_s_ contributed to reduce the A/g_s_ and to increase the transpiration rate in some monitored periods (Figure 1), the tree water status was not impaired, being even better in some sampling dates (Figure 3). Similar positive influence of shading in fruit trees water relations have been reported, either using nets [20,25] or kaolin reflective particle film [35,36,37]. Additionally, the higher values of F_v_/F_m_ under the nets during the midday period (Figure 2) indicate lower photoinhibitory damages [38] when the PPFD reaches the peak, probably associated with enhanced photosystem II (PSII) photochemistry efficiency and lower thermal energy dissipation from PSII-associated chlorophyll antennae [39]. In fact, the mean PPFD in the region during the midday period (usually above 1800 µmol m^−2^ s^−1^) is highly far above the light saturation point for photosynthesis in ‘Golden Delicious’ cultivar (1093 µmol m^−2^ s^−1^, 40). Above this point, the excessive light directly affects the oxygen-evolving complex and inactivates the PSII reaction centers, which can cause photoinhibition [40]. Moreover, photoinhibition severity is also influenced by the superimposition of other environmental stresses (expectantly higher in uncovered trees), which increase the imbalance between the damage and repair of PSII, since the produced reactive oxygen species (ROS) suppress the synthesis of proteins de novo [41]. These results are consistent with some previous studies on sunny days under hot and dry climates, which reported a positive influence on the leaf gas exchange and photochemistry processes, either using nets [24,25,42,43,44] or kaolin technology [37,38,45,46,47].

The midday depression of photosynthesis is a common phenomenon in C3 plants, including apple trees, due to the stomatal closure and/or photoinhibition caused by the accentuation of stressful conditions [42,48,49,50]. Interestingly, the likely attenuation of stressful conditions under the netting systems also alleviated the midday depression of photosynthesis, due to both lower stomatal and non-stomatal limitations. (Figure 1) Such responses of fruit trees under shading conditions are supported by the literature [42,44,46], as light, temperature and VPD are usually more determinant for midday depression of photosynthesis than tree water status and soil water availability [49,51].

Interestingly, some differences were recorded on leaf gas exchange traits between nets (Figure 1). During the morning periods of 3 July and 7 September, black net trees presented higher g_s_ and A than grey net plants, due to the higher radiation restriction caused by the black net, what might contribute to increase the attenuation of stressful conditions. Meanwhile, during the midday periods black net trees lose the capacity to maintain higher g_s_ and A relatively to grey net plants, suggesting that the differences in the intercepted radiation might not be enough to change the microclimate during this period. Moreover, on 3 August only grey net trees exhibited higher g_s_ and A than the uncovered trees, suggesting that the shading caused by the black net reduces the CO_2_ assimilation capacity when the PPFD is low, as recorded in this morning period (around 1000 µmol m^−2^ s^−1^, above the net). In the same way, reduction of A was recorded under hail nets on cloudy conditions [19]. These data suggest that oscillations in PPFD levels across the daytime and during the growing season determine the influence of each net color in the photosynthetic response.

Following a typical shade adaptation, the leaf Chl_(a+b)_ concentration and Chl_(a+b)_/Car ratio were higher under the nets (Table 2). However, Chl_a/b_ was not affected, suggesting that factors other than shading might have affected photosynthetic pigments. Indeed, the Chl_(a+b)_/Car ratio is considered a sensitive indicator of photooxidative damage [52]. As the Car concentration was not affected by the nets (Table 2), the higher Chl_(a+b)_/Car ratio under the nets was a reflex of higher Chl_(a+b)_ concentrations. Since chlorophylls are highly susceptible to environmental stresses [52], these data confirmed that shaded trees experienced reduced stressful conditions. The higher Chl_(a+b)_ concentration of netting trees might also be determinant to the higher photosynthetic rates recorded. Shaded leaves are also usually thinner than sun exposed leaves, to improve the light harvesting efficiency [53], what is consistent with the reduced leaf thickness observed in the trees under the black net in September, as inferred by LMA data (Figure 3). Similar shade adaptations were recorded in trees under net systems, especially in the darker ones [6,13,19,44], and sprayed with kaolin [36,37].

The general higher concentration of minerals in leaves under the nets across the vegetative season (Figure 4 and Figure 5) could be, in part, promoted by the water movement associated with higher g_s_ (Figure 1) [26]. However, as not all the nutrients were affected in the same way by the nets, the observed responses might be related to changes in specific nutrient metabolic processes. This behavior suggests a selective uptake and use of minerals according to the apple trees physiological response to the potential microclimate changes. In line with the improved photosynthetic responses of covered trees (Figure 1), N, Mg, Fe and Cu are known to be connected with different processes related with photosynthesis. Nitrogen is an essential constituent of proteins and chlorophylls [54], Mg is the central atom of the chlorophyll molecule, and fluctuations in its levels in the chloroplast regulate the activity of key photosynthetic enzymes [55], while Fe, besides to be required for chlorophyll synthesis and chloroplasts development, is also an important enzymatic cofactor [26], and Cu, besides the important role in redox systems, is also found in electron carrier proteins, being more than half of the Cu in plants found in chloroplasts [56]. However, these data contradict the findings of Mészáros et al. [21], who found that nets did not affected Mg and limited the uptake of N into leaves, possibly explained by a higher soil water content under the hail nets, enabling higher N leaching into lower soil layers. On the other hand, the same authors reported a positive effect of nets on micronutrients accumulation into leaves, namely on Fe, Mn and Zn.

Although the recorded differences in yield were not significantly different (Figure 6), it is possible to infer a positive influence of the nets in apple trees productivity. On the literature, the nets influence in yield is usually contradictory, varying from increase, the absence of influence to a decrease, depending largely on nets colour and environmental conditions [2,6,18,21,22,27]. In the present study, the absence of significant differences, despite the better physiological performance exhibited by the covered trees, can be due to a higher investment of trees in vegetative growth, as a typical shade avoidance response [57]. Iglesias and Alegre [18] reported no influence in yield, while the trees vigour increased by a black net in a warm region with sunny days and high radiation levels. Nonetheless, in their study the photosynthetic performance of trees was not evaluated. The increase in vigour of covered trees was also reported in other works [18,19,58,59]. In fact, the likely reduction in red:far-red ratio boosts auxin biosynthesis, leading to increased growth [57]. The higher N concentration in shaded trees (Figure 4) cloud also promotes higher vegetative growth and development [54]. The tendency for higher yields in shaded trees is strengthened by the 87% and 93% higher mean fruit weight in grey net and black net, respectively, in relation to the uncovered control (Figure 6). Moreover, fruit size might be the target parameter to evaluate trees productivity, as is considered the most interesting trait for the fruit industry [20]. In line, it is common to found higher fruit size or weight in shaded trees [6,19,20,43], although in some situations, as in the present study, no changes in yield were recorded [6,19]. The increased fruit grow in this study might be related with the improved tree water status, increased photosynthetic capacity and the reduced stress-induced damages that might allowed shaded trees to allocate more photoassimilates into the fruits instead of repairing stress-induced damages. The netting influence might also delay fruit maturity giving more time to the fruit to grow, as reported by Lopez et al. [20].

## 4. Materials and Methods

### 4.1. Plant Material and Growth Conditions

The experimental trial took place in Fonte Longa, Carrazeda de Ansiães, Northeast Portugal (41°13′07.1″ N 7°16′29.7″ W; 800 m above sea level), during the 2017 growing season, on a commercial 8-years-old apple orchard (*Malus* × *domestica* Borkh cv. ‘Golden Delicious’), grafted on MM106 rootstock and trained as central leader. The rows were oriented from NE to SW, and spaced 4 m. Tree spacing in the rows was 1.40 m, which represents a density of 1750 plants per hectare.

Climate in the region is typically Mediterranean with some Atlantic influence. According to Köppen-Geiger classification, the study site is classified as Csb, a temperate climate with hot and dry summers and rainy winters [60]. The region is also characterized by sunny days and high radiation levels over the most part of summer and is particularly vulnerable to hail events. In 2017, from June to September, the precipitation was 7.9, 7.1, 3.7 and 0 mm, respectively, and the average temperatures were 21.3, 21.8, 21.7 and 17.8 °C, respectively. In the monitored days no rainfall events were recorded, while the minimum and maximum temperatures were 18.3 and 32.2 °C on 3 July, 14.1 and 29.78 °C on 3 August, and 12.3 and 29.3 °C on 7 September.

The soil where the orchard is planted is classified in the group of Distric Leptosols (IUSS Working Group WRB, 2014) and is derived from schist. Soil analysis, from samples taken at the 0–0.2 m layer and carried out at the beginning of the study revealed a sandy-loam textured soil (67.4% sand, 18.5% silt, and 14.1% clay). Soil total organic carbon (C) was 1.1 g kg^−1^ (incineration method), pH (soil:water, 1:2.5) was 5.5, extractable phosphorous (P_2_O_5_) and potassium (K_2_O) (Egner-Riehm) were 24.5 and 148.0 mg kg^−1^, respectively, and extractable boron (B) (Azomethine-H) was 2.0 mg kg^−1^. Exchangeable potassium (K), sodium (Na), calcium (Ca) and magnesium (Mg) (ammonium acetate, pH 7) were 0.6, 0.5, 4.2 and 1.0 cmol_c_ kg^−1^, respectively. The ground of the orchard was managed with a cover of self-reseeding annual legumes, which was destroyed in May with a rotary slasher and left on the ground as a mulch. The orchard was irrigated and protected from pests and diseases according to the best practices of the region.

### 4.2. Experimental Design and Monitoring

During the years before the experiment, all trees were managed equally to guarantee the uniformity of plant development. From May 2017 (after flowering and pollination ceased) until October 2017 (after harvest), a 4 ha of the orchard was covered by a grey anti-hail net (GN) and a same area was covered by a black anti-hail net (BN). Surrounding 4 ha orchard rows were utilized as uncovered control plots (U). The nets (Benihail, Beniplast Benitex, Valencia, Spain) were made of polyethylene, with threads of 0.28 cm of thickness, with a cell size of 3 × 7.4 mm and was applied without overlapping with a small inclination towards the centre of the inter-row. In grey net the threads were distributed as 50% black (vertical threads) and 50% white (horizontal threads). The effect of nets on the interception of solar radiation was measured as a percentage of total above canopy photosynthetic photon flux density (PPFD) and ultraviolet (UV) irradiance, using a Ceptometer (Decagon Sunfleck Ceptometer, Pullman, WA, USA) and an ILT1400A radiometer (International Light Technologies, Peabody, Baltimore, MA, USA) with a photodetector SEL005/WBS320, respectively. The sensors were placed in the middle of two rows, positioned 1 m above ground level. Ten readings per treatment were taken at one-hour interval, between 9.00 and 18.00 (local time) several times in each season (July–August), after shoot growth had stopped, on both sunny and cloudy days. On sunny days an average PPFD reduction of 22% in the black net and 14% in the grey net, while for the UV irradiance a drop of 24% in the black net and 16% in the grey net were observed. On cloudy periods, here defined with PPFD lower than 500 μmol photons m^−2^ s^−1^, the reductions reached maximum values of 42 and 33% for PPFD and UV and 32 and 22% in the black and grey nets, respectively.

The experimental trial included 3 randomized blocks per treatment (U, GN, and BN), each one with 4 rows, with 30 apple trees per row. From the 2 central rows 4 trees, 2 per row, were randomly selected to be monitored. In sum a total of 12 apple trees per treatment were used.

All the physiological and biochemical measurements done at leaf level were taken in healthy, fully expanded, mature leaves. The leaf relative water content, sclerophylly indexes, gas exchange and chlorophyll *a* fluorescence measurements were taken periodically, while the samples for leaf biochemical analysis were collected at 3 August. To determine the nutritional status of the trees, a pool of leaf samples per block were taken in 3 periods during the growing season (July, August and September). To evaluate glomalin-related soil proteins concentration a composite of 4 soil samples (0–20 cm) per block was taken beneath the tree crown.

### 4.3. Glomalin-Related Soil Proteins

After drying and sieving (2 mm mesh), the soil samples were submitted to determinations of total and easily extractable glomalin-related soil proteins (GRSP) according to the methodology of Wright and Updahyaya [61].

### 4.4. Leaf Gas Exchange and Chlorophyll a Fluorescence

Leaf gas exchange measurements were performed using a portable IRGA (LCpro+, ADC, Hoddesdon, UK), operating in the open mode. Measurements were performed on sun exposed leaves in two periods, morning (mo, 9:00–10:00 local time) and midday (md, 13:00–14:00 local time) of cloudless days, under natural irradiance and environmental conditions. Net photosynthetic rate (A, μmol CO_2_ m^−2^ s^−1^), stomatal conductance (g_s_, mmol H_2_O m^−2^ s^−1^) and the ratio of intercellular to atmospheric CO_2_ concentration (C_i_/C_a_) were estimated using the equations developed by von Caemmerer and Farquhar [62]. Intrinsic water use efficiency was calculated as the ratio of A/g_s_ (μmol mol^−1^) [63]

Chlorophyll a fluorescence variables were measured in the same leaves and environmental conditions used for gas exchange measurements, with a pulse-amplitude-modulated fluorometer (FMS 2, Hansatech Instruments, King’s Lynn, UK). Prior to the measurements, a small part of the leaves was dark-adapted for 30 min using dark-adapting leaf-clips. After this period, the minimal fluorescence (F_o_) was measured when all photosystem II (PSII) reaction centres are open using a low intensity pulsed measuring light source. The maximal fluorescence (F_m_) was measured when all PSII reactions centres are closed during a pulse saturating light (0.7 s pulse of 15,000 µmol photons m^−2^ s^−1^ of white light). The difference between these two levels (F_m_ − F_o_) is called variable fluorescence (F_v_). Maximum quantum efficiency of PSII was calculated as F_v_/F_m_ = (F_m_ − F_o_)/F_m_ [31]. Following, F_v_/F_m_ estimation, after a 20 s exposure to actinic light (1500 µmol m^−2^ s^−1^), light-adapted steady-state fluorescence yield (F_s_) was averaged over 2.5 s, followed by exposure to saturating light (15,000 µmol m^−2^ s^−1^) for 0.7 s to establish F’_m_. The sample was then shaded for 5 s with a far-red light source to determine F’_o_. From these measurements were calculated the capture efficiency of excitation energy by open PSII reaction centers (F’_v_/F’_m_ = (F’_m_ − F’_o_)/F’_m_) and effective quantum efficiency of PSII (ΦPSII = ΔF/F’_m_ = (F’_m_ − F_s_)/F’_m_) [64,65]. Due to a problem in the fluorometer, it was not possible to assess the chlorophyll a fluorescence responses in August. The measurements of leaf gas exchange and chlorophyll fluorescence were made on summer days with photosynthetic photon flux density of 1418 ± 80 μmol photons m^−2^ s^−1^ and 1853 ± 55 μmol photons m^−2^ s^−1^ on 3 July during morning and midday periods, respectively, 948 ± 41 μmol photons m^−2^ s^−1^ and 1917 ± 32 μmol photons m^−2^ s^−1^ on 3 August during morning and midday periods, respectively, and 1390 ± 69 μmol photons m^−2^ s^−1^ on 7 September during the morning period.

### 4.5. Leaf Water Status and Sclerophylly Indexes

After the leaf gas exchange and chlorophyll *a* fluorescence measurements, the same leaves were collected and immediately placed into air-tight containers and the following parameters were examined: fresh weight (FW; g); fresh weigh at full turgor (TW; g), measured after immersion of leaf petioles in demineralized water for 24 h in the dark at 4 °C; leaf area (LA), measured with an LI-3100 leaf area meter (Li-Cor, Lincoln, NE, USA); and dry weight (DW; g), measured after drying in a force-draft oven at 60 °C to a constant weight. Further, it was calculated the leaf relative water content (RWC = (FW − DW)/(TW − DW) × 100%), the leaf mass area (LMA = DW/LA, g m^−2^) and the density of foliar tissue (D = DW/FW, g kg^−1^).

### 4.6. Leaf Biochemical Analysis

For leaf biochemical analysis, the harvested leaves were immediately frozen in liquid nitrogen and stored at −80 °C until be analysed. To express the metabolites by dry mass, a representative sample of each analysed leaf was evaluated in fresh and after drying at 60 °C until constant weight. Chlorophylls and carotenoids were extracted with acetone/water (80/20, *v*/*v*). Chlorophyll a (Chl_a_), chlorophyll b (Chl_b_) and total chlorophyll (Chl_(a+b)_) were determined according to Arnon [66] and Sesták et al. [67] and total carotenoids (Car) according to Lichtenthaler [68] and expressed as mg g^−1^ DW.

Total soluble sugars (TSS) were extracted according to Irigoyen et al. [69], by heating the samples in ethanol/water (80/20, *v*/*v*) during 1 h, at 80 °C. Then, the soluble fractions were separated from the solid fraction.

Total soluble proteins (TSP) were quantified using the method of Bradford [70], using bovine serum albumin as a standard, and expressed as mg g^−1^DW. Then, total thiols (-SH) in soluble proteins extract were assessed according to Ellman [71], using an extinction coefficient of 13,600 M^−1^ cm^−1^, and being expressed as nM mg^−1^DW.

### 4.7. Leaf Ionome

Leaves were collected from the middle of current season shoots of the four quadrants around the tree canopy. The samples were then oven-dried at 70 °C and ground. Tissue analyses were performed by Kjeldahl (N), colorimetry (B and P), flame emission spectrometry (K) and atomic absorption spectrophotometry (Ca, Mg, Cu, Fe, Zn, and Mn) methods [61].

### 4.8. Yield and Fruit Weight

Harvest was performed when fruit achieved their commercial maturity. For each tree total yield (kg tree^−1^) was determined. From each tree were randomly selected 10 fruits to determine average fruit fresh weight (g fruit^−1^).

### 4.9. Statistical Analysis

All statistical calculations were performed using the software program SPSS for Windows (V22.0). After testing for ANOVA assumptions (homogeneity of variances with the Levene’s mean test, and normality with the Kolmogorov-Smirnov test), statistical differences were evaluated by one-way analysis of variance (ANOVA), followed by the pot-hoc Tukey’s test (*p* < 0.05). For statistical analysis of RWC arcsine transformation was performed in percentage data.

## 5. Conclusions

Under the conditions of the study, the microclimatic changes created by the grey and black nets contributed to reduce the orchard vulnerability to the highly irradiances far above the apple tree light saturation point and the hot and dry summer season. This assumption is taken by the reduced glomalin related-soil proteins accumulation in the soils underneath the nets, and, in covered trees, by the improvement of water and mineral status indices and photosynthetic responses, the reduction in the oxidative damages (traduced by the higher concentrations of chlorophylls) and the improvement of fruit weight.

The great difference between the two nets laids on the photosynthetic activity. Black-net was more efficient on sunny days, while the grey-net stoodout under cloudy conditions. The occurrence of different environmental conditions across the fruit growing season led to similar responses to both nets in terms of yield and fruit weight.

Currently, the major challenge in fruit production is to optimize the sustainable use of limited natural resources by minimizing the negative effects of abiotic stresses in trees. In this regard, the use of netting systems proved to be effective in improving ‘Golden Delicious’ apple trees performance under the climatic conditions of this study. These findings contribute to the need for specifying recommendations regarding the apple orchard management in a context of climate change, especially in areas in which irrigation water is scarce and high levels of irradiance and hailstorms occur.

## Figures and Tables

**Table 1:** Total and easily extractable glomalin-related soil proteins (mg g^−1^ DW) in soils uncovered (U), and under grey (GN) and black nets (BN).

	Total Extractable Glomalin-Related Soil Proteins	Easily Extractable Glomalin-Related Soil Proteins
U	2.12 ± 0.31 ^a^	0.736 ± 0.082 ^a^
GN	0.751 ± 0.073 ^b^	0.418 ± 0.048 ^b^
BN	0.461 ± 0.024 ^b^	0.289 ± 0.057 ^b^
Sig	***	***

Values are means ± SE. Different letters demonstrate significant differences between treatments (*** *p* < 0.001).

**Table 2:** Leaf biochemical analysis of plants uncovered (U) and under grey (GN) and black net (BN). Total soluble sugars (TSS, mg g^−1^ DW), total chlorophylls (Chl_(a+b)_, mg g^−1^ DW), chlorophyll a/chlorophyll b ratio (Chl_a/b_), total carotenoids (Car, mg g^−1^ DW), total chlorophylls/ total carotenoids ratio (Chl_(a+b)_/Car), total soluble proteins (TSP, mg g^−1^ DW) and total thiols (-SH, µmol g^−1^ DW).

	U	GN	BN	Sig
TSS	84.2 ± 2.5	79.9 ± 6.3	86.8 ± 5.6	n.s.
Chl_(a+b)_	3.33 ± 0.28 ^b^	4.38 ± 0.23 ^a^	4.29 ± 0.18 ^a^	*
Chl_a/b_	2.83 ± 0.18	2.86 ± 0.10	2.68 ± 0.18	n.s.
Car	2.00 ± 0.19	1.87 ± 0.17	1.77 ± 0.132	n.s.
Chl_(a+b)_/Car	1.67 ± 0.09 ^b^	2.36 ± 0.14 ^a^	2.45 ± 0.11 ^a^	**
TSP	9.92 ± 0.33	10.1 ± 0.5	9.36 ± 0.30	n.s.
-SH	2.49 ± 0.22	2.48 ± 0.09	2.27 ± 0.13	n.s.

Values are means ± SE. Different letters demonstrate significant differences between treatments (n.s.—not significant; * *p* < 0.05, ** *p* < 0.01).

## Data Availability

Data is contained within the article.
